# Challenges and Misinterpretations of Cohen's Kappa in Agreement Studies in Ophthalmology

**DOI:** 10.1111/vop.70208

**Published:** 2026-07-19

**Authors:** Malwina Ewa Kowalska, Niklas Holz, Simon Anton Pot, Antonella Rampazzo, Sonja Hartnack

**Affiliations:** ^1^ Ophthalmology Section, Vetsuisse Faculty University of Zurich Zurich Switzerland; ^2^ Section of Epidemiology, Vetsuisse Faculty University of Zurich Zurich Switzerland; ^3^ Centre for Veterinary Systems Transformation and Sustainability, Clinical Department for Farm Animals and Food System Science University of Veterinary Medicine Vienna Vienna Austria

**Keywords:** gonioscopy, inter‐rater, kappa coefficient, maxKappa, reliability studies

## Abstract

**Objective:**

Cohen's kappa is widely used to measure rater agreement on binary outcomes and performs well when outcome prevalence is relatively balanced. This paper aims to explain the challenges encountered with Cohen's kappa, and to provide guidance to veterinary ophthalmologists on the usage and interpretation of kappa values.

**Animals Studied:**

Article based on an existing dataset in which two examiners graded iridocorneal angle abnormalities in 60 client‐owned dogs during pre‐breeding examinations.

**Procedure:**

Following the 2022 ECVO‐HED (European College of Veterinary Ophthalmology—Hereditary Eye Diseases) gonioscopy grading scheme and its general recommendations, eyes were classified into two categories—“breeding‐YES” (not affected, mildly or moderately affected) or “breeding‐NO” (severely affected). Inter‐examiner agreement was separately assessed for real (60 left and 58 right eyes) and simulated data (manually balanced left eye dataset with unchanged agreement between examiners).

**Results:**

While the two examiners classified 52/60 left and 51/58 right eyes into the “breeding‐YES” category, they disagreed on 7/60 left and 1/58 right eyes, with resulting kappa values of 0.18 and 0.91 for the left and right eyes, respectively. After balancing the data, with both examiners classifying 27/60 left eyes into the “breeding‐yes” category, but still disagreeing on 7/60 eyes, the kappa value increased to 0.77.

**Conclusions:**

Readers should be cautious when comparing kappa values across studies with different underlying prevalence and bias. Under non‐ideal data distribution, additional statistical indices like prevalence‐ and bias index, prevalence‐and‐bias‐adjusted kappa (PABAK), and maxKappa, do not replace Cohen's kappa, but assist in interpreting it.

## 1 Introduction

Cohen's kappa, introduced by Dr. Jacob Cohen in 1960 [1], is commonly used in the medical field to assess the degree of rater agreement, also named inter‐rater (inter‐examiner) reliability. The use and interpretation of Cohen's kappa present certain challenges, which were identified and published as early as the 1980s [2, 3].

This article aims to outline the challenges associated with Cohen's kappa and to provide guidance on its use and interpretation to veterinary ophthalmologists. These approaches do not ‘correct’ kappa but provide essential context for its interpretation.

The article is organized as follows: (2) introduction to kappa statistics, (3) application of kappa with real and simulated data, (4) presentation of potential adjustments to kappa reporting, and (5) discussion of kappa application and interpretation.

## 2 Introduction to Kappa Statistics

Cohen's kappa assesses the agreement between two raters or examiners when classifying subjects into binary outcomes (e.g., positive/negative, yes/no, or pass/fail). Simply stating the percentage of observed agreement, i.e., saying that the two examiners agreed in 90% of cases, can be misleading, as some of the observed agreement may have occurred due to chance. For example, if two coins were simultaneously flipped, the expectation would be that—purely by random chance—both coins would “agree” by both showing either heads or tails in about half of the flipped turns.

**FIGURE 1:**
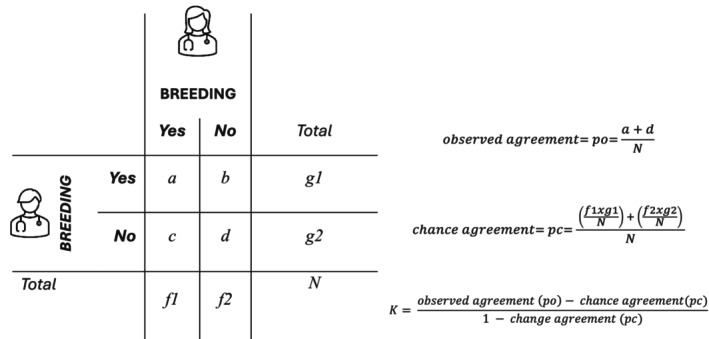
Calculation of Cohen's kappa.

For obvious reasons, examiners are not expected to classify subjects only randomly, but the possibility exists that some inter‐examiner agreement is due to chance. Cohen's kappa aims to present a chance‐adjusted measure of agreement between two examiners, i.e., the agreement between examiners that is not a result of chance alone. Figure 1 demonstrates how kappa can be determined using the frequencies in a 2 × 2 table, in which N subjects were cross‐classified into breeding “yes” or “no”. The non‐agreeing, discordant ratings are found in cells b and c. The agreeing, concordant ratings are found in cells a and d. The sum of a and d, divided by the total number N, indicates the observed agreement po, which is the proportion or probability of agreement. It can be alternatively presented as a percentage when multiplied by 100. To determine the hypothetical probability of chance agreement ***pc***, we would first multiply all eyes classified as “breeding yes” (respectively as “breeding no”) by both examiners. This is done by multiplying the total of the rows and columns f1×g1, respectively f2×g2, dividing both products by the total number N, and summing up both fractions. To finally obtain the probability of chance agreement pc, the sum of both fractions is also divided by N (Figure 1).

Cohen's kappa, K is obtained by subtracting the chance agreement pc from the observed agreement po, divided by 1−pc.

Kappa is always smaller than po, unless there is a perfect agreement between the examiners, where both K and po are equal to 1. Kappa usually takes values between 0 and 1, but negative values are also possible when the agreement is worse than what would be expected by chance.

Several scales with different cut‐off values have been developed by Cohen [1, 4, 5, 6, 7] and others to interpret the calculated kappa value (e.g., good, fair, etc.). Caution should be exercised when interpreting kappa based on various cut‐off values, as these are somewhat arbitrary.

Cohen's kappa was developed on the assumption that the same subjects are classified into two different categories by two examiners, i.e., there are two ratings for each subject. These two ratings for the same subject can be considered as paired or dependent samples. Kappa is also based on the assumption that each examiner only rates each subject once, i.e., that the paired ratings are independent across subjects. In ophthalmology, however, data are often clustered within subjects (i.e., both eyes **from an individual** are rated). While this differs from the traditional “lack of independence” where an examiner might be influenced by a previous rate, it creates a within‐cluster dependency. As noted by Sim and Wright, ignoring such dependencies can lead to elevated Type I errors (i.e., lower *p*‐value, and consequently, significant results when truly they are not) and narrower confidence intervals (i.e., more precise results than we actually have) [8]. The degree of this effect depends on the correlation (similarity) between the eyes. The higher the positive correlation, the more the inflation of precision (low *p*‐value and narrow confidence intervals). The assumption of independence is strictly met only when each subject contributes a single eye to the kappa calculation. However, including only one eye per subject presents a dilemma: either the data must be reported separately for each eye, or half of the available data must be discarded. Solutions to this dilemma will be briefly presented in the discussion.

Further extensions of kappa have been developed for situations with more than two examiners (Fleiss kappa) or more than two rating categories (weighted kappa) [8].

## 3 Application of Kappa With Real and Simulated Data

In pre‐breeding assessments, the ECVO‐HED (European College of Veterinary Ophthalmology—Hereditary Eye Diseases) 2022 gonioscopy grading scheme (ECVO Manual, Chap. 6.1) [9] is typically employed by a single ophthalmologist. The study conducted by Holz et al. [10] aimed to evaluate inter‐examiner agreement using this grading scheme. Two ophthalmologists with similar clinical expertise independently assessed 60 dogs using the ECVO‐HED 2022 gonioscopy grading scheme and its guidelines for evaluating the iridocorneal angle. Where feasible, both eyes of each dog were examined, with random assignment of the sequence of examiners performing the first gonioscopy on the first eye. The examiners were masked to the ratings assigned by the second examiner for the duration of the study. To calculate Cohen's kappa, the detailed ECVO‐HED grading results were consolidated into two final breeding recommendation categories for the left and right eye separately: “breeding yes” (encompassing the Iridocorneal Angle Abnormality (ICAA) grades ‘unaffected’, ‘mildly affected’, and ‘moderately affected’) and “breeding no” (ICAA grade ‘severely affected’).

Following the FAIR (findable, accessible, interoperable, reusable) principles [11], the dataset is available at the Open Science Framework (Holz et al. [10]). The analysis was performed in the freely available software R version 4.4.1 [12]. Cohen's kappa was calculated using the command epi.kappa() available in the package epiR [13]. The code used for the analysis is presented in S1. Since maxK is not a standard output of the R epiR package, the calculation method is also provided.

In the following study, the eyes were interpreted separately. Particularly in the case of gonioscopy clustering within subjects is high, knowledge of one eye can inform the rating of the second eye. Table 1 illustrates the left eye data scenario, where examiner 1 graded 97% of the eyes (58 out of 60) as “breeding yes” while examiner 2 graded 88% of the eyes (53 out of 60) as “breeding yes”. Both examiners agreed on a “yes” for 52 eyes and on a “no” for 1 eye, resulting in agreement (concordance) between examiners for 53 out of 60 eyes. Consequently, the observed agreement po is 88%. The examiners disagreed (discordance) in 7 out of 60 eyes (12%). The resulting kappa value is 0.18, which indicates a slight agreement according to the Landis and Koch [4] and a poor agreement according to Altman [5] and to Fleiss et al. [6].

**TABLE 1:** Cross‐classified ECVO‐HED ratings for the left eyes of 60 dogs by two examiners (based on Holz et al. [10]).

OS			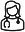		Total	Cohen's kappa (95% CI)
			Breeding			
			*Yes*	*No*		
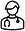	**Breeding**	***Yes***	52	6	58 (97%)	0.18 (−0.02 to 0.38)
		***No***	1	1	2 (3%)	
Total			53 (88%)	7 (12%)	60	
Total agreement: 52 + 1 = 53						

*Note:* Data in this table represent a binary collapse of the original ECVO‐HED categories (e.g., “breeding‐Yes”/“breeding‐No”) rather than the original multi‐level grading scale.

Table 2 presents the right eye data scenario, where examiner 1 graded 88% of the eyes (51 out of 58) as “breeding yes” and examiner 2 graded 90% of the eyes (52 out of 58) as “breeding yes”. Both examiners agreed on a “yes” for 51 eyes and on a “no” for 6 eyes, resulting in agreement (concordance) between examiners for 57 out of 58 eyes. Consequently, the observed agreement po is 98%. The examiners disagreed (discordance) in 1 out of 58 eyes (2%). The resulting kappa value is 0.91, which indicates an almost perfect agreement according to Landis and Koch [4], a very good agreement according to Altman [5] and an excellent agreement according to Fleiss [6].

**TABLE 2:** Cross‐classified ECVO‐HED ratings for the right eyes of 58 dogs by two examiners (based on Holz et al. [10]).

OD			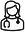		Total	Cohen's kappa (95% CI)
			Breeding			
			*Yes*	*No*		
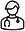	**Breeding**	***Yes***	51	0	51 (88%)	0.91 (0.66 to 1.17)^a^
		***No***	1	6	7 (12%)	
Total			52 (90%)	6 (10%)	58	
Total agreement: 51 + 6 = 57						

^a^ Confidence intervals are based on mathematical calculations and therefore may exceed the possible range for kappa: −1 to 1. Interpret estimates only within these logical bounds. Data in this table represent a binary collapse of the original ECVO‐HED categories (e.g., “breeding‐Yes”/“breeding‐No”) rather than the original multi‐level grading scale.

When comparing the kappa values for the left and right eyes, which are 0.18 and 0.91, respectively, and assuming that no plausible biological or pathophysiological cause for the discrepancy in agreement between these side‐specific ratings exists, the challenge lies in explaining the large difference in kappa values between the left and right eyes. Soon after the development of Cohen's kappa, problems in interpretation and potential misuses of Cohen's kappa have been described and attributed to prevalence and bias [2, 14].

The separate analysis of the left and right eye in the publication was done to illustrate bias and prevalence effects on kappa. Presenting separate results is not the ideal approach for estimating kappa or its confidence intervals. Solutions for using clustered within‐subject data will be briefly explored in the discussion.

### 3.1 Prevalence

Prevalence greatly influences kappa values. In the study by Holz et al. [10], the “prevalence” would be the proportion of “breeding yes” or “breeding no” ratings in the examined eyes. The closer these proportions are to 0.5, i.e., as many “yes/yes” as “no/no” ratings, the higher the kappa value will be. This is demonstrated in Table 3. Here, the dataset for the left eyes from Holz et al. [10] (Table 1) was manually balanced to have equal proportions of concordant ratings (“yes/yes” and “no/no”), whereas the agreement between examiners was left unchanged, with the same observed agreement ***po*** of 88%—corresponding to 53 out of 60 eyes. Observed agreement was fixed at 88% to isolate the effect of prevalence and bias on kappa independently of agreement magnitude. As a result of a more balanced agreement with 27 “yes/yes” and 26 “no/no” concordant ratings, the kappa value now increased to 0.77 (Table 3), compared to the previous kappa value of 0.18 for 52 “yes/yes” and 1 “no/no” concordant ratings (Table 1). The kappa value obtained by the simulated data set is very close to the PABAK (prevalence‐ and bias‐adjusted kappa), which will be presented in the next paragraphs. Thus, one important factor that affects kappa values is the prevalence, which corresponds here to the proportion of “breeding yes” or “breeding no” ratings in the examined eyes.

**TABLE 3:** Simulated cross‐classified ECVO‐HED ratings for the left eyes of 60 dogs by two examiners.

OS			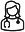		Total	Cohen's kappa (95% CI)
			Breeding			
			*Yes*	*No*		
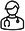	**Breeding**	***Yes***	27	6	33 (55%)	0.77 (0.52 to 1)
		***No***	1	26	27 (45%)	
Total			28 (47%)	32 (53%)	60	
Total agreement: 27 + 26 = 53						

*Note:* Note that identical observed agreement can yield widely different kappa values. Data in this table represent a binary collapse of the original ECVO‐HED categories (e.g., “breeding‐Yes”/ “breeding‐No”) rather than the original multi‐level grading scale. Green cells ('a' and 'd') denote adjusted values from the original Table 1 to present a balanced prevalence scenario.

**FIGURE 2:**
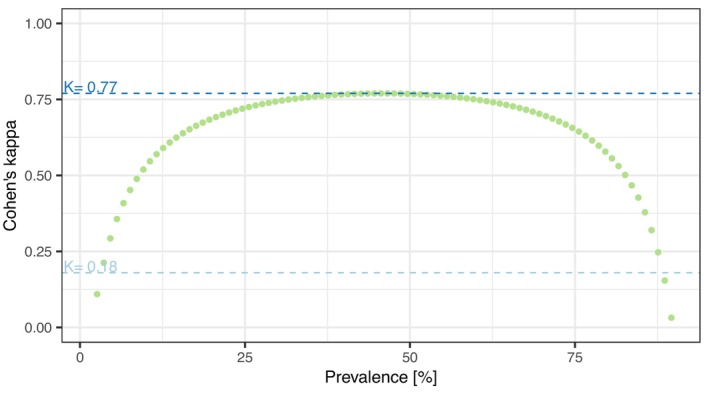
Kappa values for simulated data with an 88% observed agreement and varying prevalence.

The effect of the varying prevalence on kappa is displayed in Figure 2. In this simulated data set, we assume an observed agreement po of 88% (equal to 12% discordant ratings) between examiners and vary the proportion of the concordant “yes/yes” and “no/no” ratings, i.e., the prevalence. The kappa values are highest when the prevalence is close to 50%, and most stable with prevalences between 25% and 75%.

### 3.2 Bias

In addition to the balance between concordant ratings (prevalence), the balance between discordant ratings (i.e., the proportion of “yes/no” and “no/yes” ratings) is relevant and can also affect kappa values. This imbalance is referred to as bias. In the study by Holz et al. [10], for the left eye scenario, there were six “yes/no” ratings (cell *b*. Table 1) and one “no/yes” rating (cell *c*. Table 1), whereas in the right eye scenario, there was only one “no/yes” rating. The primary factor accounting for the disparity in kappa values between right and left eyes in the study by Holz et al. [10] is the difference in the proportion of discordant ratings, which was 12% for left eyes compared to 2% for right eyes.

**FIGURE 3:**
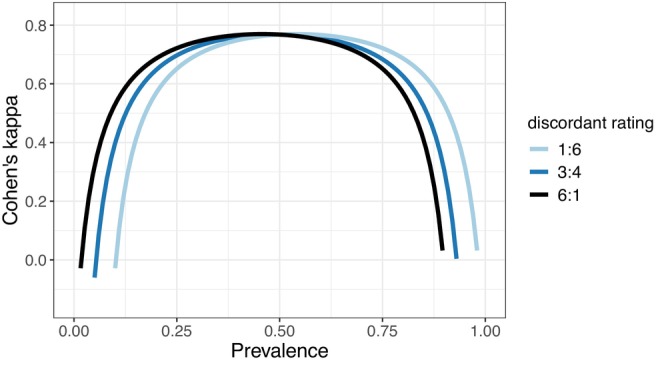
Kappa values for simulated data with an 88% observed agreement and varying bias.

The proportion of discordant ratings (bias) shifts the curve of possible kappa values horizontally (Figure 3). In the simulated data set for the left eye, we show three examples of discordant ratings (cells b/c): original proportion (6/1), reversed (1/6), and balanced (3/4). Note that kappa values are the most stable (not influenced by bias), with a prevalence of 50%.

## 4 Potential Adjustments to Kappa Reporting

To better understand the effects of prevalence and bias on kappa values, several supplementary indices—such as positive and negative agreement, the Prevalence Index (PI), the Bias Index (BI), and the maxKappa—have been developed. Unlike the Prevalence‐and‐Bias‐Adjusted Kappa (PABAK), which is a truly adjusted agreement index, PI, BI, and maxK function as descriptive metrics. They are intended to be reported alongside Cohen's kappa as safeguards alerting the reader when kappa may not be the most representative index for the data.

Positive agreement (*P*_pos_) and negative agreement (*P*_neg_) quantify consistency for each category separately. These are calculated as 2*a*/(2*a* + *b* + *c*) and 2*d*/(2*d* + *b* + *c*), respectively. Reporting these indices prevents a high kappa from masking poor performance in a rare but important category. In our original OS data (Table 1), *P*_pos_ was 94% but *P*_neg_ was only 22%, showing that examiners struggled to agree on “breeding no” cases. The simulated data (Table 3) showed balanced consistency with both P_pos_ and P_neg_ at 88%.

The prevalence index (PI), defined as $\mathrm{PI}=\frac{a}{N}-\frac{d}{N}$, quantifies the differences between concordant “yes” and “no” ratings [15]. Absolute PI values range from 0, indicating an equal distribution of concordant “breeding yes” and “breeding no” ratings, to 1, indicating an unequal distribution. A small PI, reflecting a prevalence close to 50%, results in high kappa values. In contrast, a large PI, indicating prevalences nearer to 0% or 100%, results in low kappa values. In the original dataset for the left eye scenario (Table 1, Holz et al. [10]), the absolute PI is 0.85. In contrast, in the simulated dataset with a more balanced distribution of “yes/yes” and “no/no” ratings (Table 3), the absolute PI is 0.02. This explains the difference in kappa values between 0.18 for the real left eye data and 0.77 for the simulated balanced left eye data set.

The bias index (BI), defined as $\mathrm{BI}=\frac{a+b}{N}-\frac{a+c}{N}$, is the extent to which examiners disagree on the proportion of discordant “yes/no” and “no/yes” ratings, with absolute values ranging from 0 to 1 [15]. A BI of 0 indicates a more balanced distribution of discordant ratings, whereas a BI closer to 1 indicates a less balanced distribution. In the study by Holz et al. [10], the BI for left eyes is 0.08, compared to −0.02 for right eyes. Both PI and BI are intended to guide the interpretation of kappa values. Caution is advised when interpreting kappa values in the presence of a high PI and/or a high BI.

To reduce the intricate effects of unequal distributions of concordant (prevalence) and discordant (bias) ratings on kappa values, prevalence‐ and bias‐adjusted (PABAK) approaches have been developed [15, 16]. Unlike kappa, PABAK reflects the ideal situation by disregarding variations in prevalence between conditions and avoiding distortions present in real‐world scenarios [17].

PABAK is defined as $\text{PABAK}=\left(2\times \mathrm{po}\right)-1$, i.e., twice the observed agreement po minus 1.

PABAK is 0.77 (2 × 0.88 – 1) in the real left eye scenario (Holz et al. [10]) and similar to the kappa in the simulated left eye data set with more balanced proportions of concordant ratings. In the right eye scenario, PABAK is 0.96. While the original kappa values vary greatly between the left and right eyes, the PABAK values for the two eyes are much closer to each other. In case there is a considerable difference between kappa and PABAK, kappa values should be interpreted with caution.

In addition to PABAK, the concept of maximum kappa (*maxK*) has been proposed. The *maxK* represents the greatest possible kappa value [14, 18], within the unchanged marginal totals (the total number of eyes scored as “breeding yes” or “breeding no” by each examiner remains unchanged). This method is used to understand the upper bound of agreement between examiners. Because marginal totals are fixed, when the difference between maximum kappa and kappa is high, individual examiner tendencies, assessment methods, or differences in used equipment may be considered a relevant source of disagreement. In case with a considerable difference between kappa and *maxK*, kappa values should again be interpreted with caution.

It has been proposed that kappa is most reliable when the marginal distributions (differences in the proportions of the same categories) are similar, for which a McNemar's hypothesis test for paired samples has been suggested as a testing method [19]. Therefore, this condition could be checked before calculating kappa. Importantly, McNemar's test does not validate kappa itself but helps identify asymmetrical marginal distributions that may distort its interpretation. A significant result (*p* < 0.05) indicates asymmetry (when examiners disagree, one is more likely to choose a particular category). A non‐significant McNemar test (*p* > 0.05; insufficient evidence for asymmetry) suggests that the calculated kappa might be a fair representation of agreement. Using the command epi.kappa(), available in the package epiR [13], next to kappa, PI, BI, PABAK, the results of the McNemar test are also presented in Table 4 and the reproducible R code in the S1.

**TABLE 4:** Rounded results obtained by the command epi.kappa in the package epiR [13] for the left and right eyes, and the simulated data set.

Breeding	Table 1: left eyes		Table 2: right eyes		Table 3: simulated data	
	Yes	No	Yes	No	Yes	No
Yes	52	6	51	0	27	6
No	1	1	1	6	1	26
	Estimate	95% CI	Estimate	95% CI	Estimate	95% CI
Observed agreement	0.88^a^		0.98		0.88	
Expected agreement	0.86		0.80		0.50	
Positive agreement	0.94		0.99		0.88	
Negative agreement	0.22		0.92		0.88	
Cohen's kappa	0.18	−0.025; 0.385	0.91	0.657; 1.169^b^	0.77	0.519; 1.018^b^
P index	0.85	0.758; 0.942	0.78	0.661; 0.891	0.02	−0.161; 0.194
B index	0.08	0; 0.176	−0.02	−0.132, 0.097	0.08	−0.095; 0.261
PABAK	0.77	0.548; 0.903	0.96	0.815; 0.999	0.77	0.548; 0.903
McNemar, *p*	0.059		0.317		0.059	
maxK	0.41		0.9		0.83	

^a^ The proportion of 0.88 is equal to a percentage of 88%.

^b^ Confidence intervals are based on mathematical calculations and therefore may exceed the possible range for kappa: −1 to 1. Interpret estimates only within these logical bounds.

In summary, the striking difference in kappa values of 0.18 and 0.91 for the left and right eye scenarios can be explained by a combination of different factors, including a higher observed agreement in the right eye (0.88 versus 0.98), unbalanced prevalence of the condition scored, and scoring bias between examiners.

## 5 Discussion of Kappa Application and Interpretation

We used a real‐life dataset collected by Holz et al. [10] to illustrate the “kappa paradox” by demonstrating a substantial difference between left and right eyes in kappa values comparing the pre‐breeding assessments of two examiners. The term “kappa paradox” was coined by Feinstein and Cicchetti (1990) [20], and describes a scenario in which the proportion of agreement is high, but kappa values are low. To address this paradox—and enable a clinically meaningful interpretation of kappa—we presented PI, BI, PABAK, and *maxK*. However, these approaches have also been criticized for not reflecting the clinical setting in which the data were generated, aiming instead at an “ideal” situation [21].

Additionally, cut‐offs like those proposed by Landis and Koch [4], Altman [5], or Fleiss [6] should be interpreted cautiously, as they may be arbitrary. As a minimum, the kappa value should always be presented in combination with the author's interpretation of the result and confidence intervals.

Conceptually, beyond the unwanted effects of prevalence and bias on kappa values, it is crucial to understand that kappa measures agreement beyond chance and does not indicate the accuracy, or closeness of ratings of both examiners to the true status. In other words, Cohen's kappa does not say whether the ratings given by the examiners are correct. Low kappa values can occur if one examiner or diagnostic test classifies subjects (eyes for pre‐breeding, in our case) with high accuracy while the other does not, or if both examiners are inaccurate. Conversely, high kappa values can result from both examiners or diagnostic tests being highly accurate or both making the same errors (e.g., false positives due to cross‐reacting agents in diagnostic tests). Similarly, examiners may follow the ECVO‐HED scheme guidelines and come up with the same grades for an individual, but both may be misinterpreting the guideline definitions in the same way.

If accuracy is of interest rather than just agreement beyond chance, advanced statistical modeling approaches using latent class models and Markov Chain Monte Carlo simulations can be employed to determine the diagnostic sensitivities and specificities of examiners or diagnostic tests. Latent class models were already endorsed by the World Organization for Animal Health (OIE) in 2013 for evaluating diagnostic tests in real‐world conditions, as reviewed by Cheung [22].

This publication does not exhaust all methods available for inter‐examiner studies; rather, it highlights which indices should be reported alongside kappa to ensure a comprehensive interpretation. For example, Gwet's AC1 has been proposed as a robust alternative to Cohen's kappa for calculating reliability. Unlike PABAK, which mathematically forces the prevalence to 50% (a scenario that may not be clinically representative of the actual population), Gwet's AC1 utilizes a method for calculating expected agreement that does not assume a balanced distribution [23, 24].

The study by Holz et al. [10] was pre‐registered, meaning that the plan for data collection and analysis was described in advance and submitted to preclinicaltrials.eu (ID: CTE0000388). In general, pre‐registration is increasingly advocated and highly recommended to ensure high research quality and mitigate the reproducibility crisis by preventing data manipulation [25]. In the presented example, a potential data manipulation could involve selective reporting and presenting only the better kappa for the right eyes (and omitting the kappa value for the left eyes).

Prevalence and bias have a larger effect on kappa than within‐subject clustering, which primarily affects the CI width. Nevertheless, because clinical data in veterinary ophthalmology are commonly collected from both eyes, researchers must employ cluster‐adjusted statistical methods to account for within‐subject clustering (inter‐eye correlation) when pooled data is used. Examples of such methods include: analytic variance correction for two eyes per subject [26], CI adjustment using a design effect based on inter‐eye correlation [27], and cluster bootstrap CI [26]. The “pooled” kappa for the presented dataset is 0.6, with a bootstrap CI of 0.33 to 0.79.

For binary outcomes, such as breeding recommendations (yes/no), a practical solution for addressing within‐subject clustering is to define an animal‐level outcome. This approach was used by Holz et al. [10], where the breeding recommendation for an individual dog was determined by the more severely affected eye (i.e., the eye with the higher ICAA grade). A study pre‐registration should detail how the inclusion of two eyes per individual will be addressed in the statistical analysis.

In conclusion, while Cohen's kappa is easily and widely used, it is important to remain mindful of its challenges to ensure a clinically meaningful interpretation of results. Therefore, when kappa is used, we advocate reporting PI, BI, *maxK*, and adjustments for prevalence like PABAK next to the kappa value with its confidence interval. This will allow an interpretation of kappa values in the specific study context. Furthermore, we should remain cautious when comparing the magnitude of kappa across data sets with different prevalence or bias [14].

## Author Contributions

**Malwina Ewa Kowalska:** conceptualization, writing – original draft, methodology, visualization, software, formal analysis, data curation, investigation. **Niklas Holz:** writing – review and editing, project administration, conceptualization. **Simon Anton Pot:** resources, supervision, writing – review and editing, conceptualization, funding acquisition, methodology. **Antonella Rampazzo:** conceptualization, funding acquisition, methodology, writing – review and editing, supervision, resources. **Sonja Hartnack:** validation, conceptualization, methodology, writing – review and editing, software, supervision.

## Funding

The authors have nothing to report.

## Disclosure

Artificial intelligence statement: The authors have not used AI to check part of the R code avaliable in the S1.

## Ethics Statement

The data used originate from the study of Holz et al., which was approved by the Federal Food Safety and Veterinary Office of Switzerland. National no. 35599; Cantonal no. ZH025/2023.

## Conflicts of Interest

The authors declare no conflicts of interest.

## Supporting information




**S1:** Challenges and misinterpretations of Cohen's kappa in agreement studies in ophthalmology.

## Data Availability

The data that support the findings of this study are openly available in OSF at https://osf.io/rdbt9/, reference number DOI 10.17605/OSF.IO/RDBT9.
