# Supplementary material for: Challenges and Misinterpretations of Cohen's Kappa in Agreement Studies in Ophthalmology
Source: Vet Ophthalmol. 2026 Jul 19;29(5):e70208. doi: 10.1111/vop.70208 (PMC13381809; doi:10.1111/vop.70208)
Supplement: Supplementary file 1 — S1: Challenges and misinterpretations of Cohen's kappa in agreement studies in ophthalmology. [file VOP-29-0-s001.docx]

Challenges and misinterpretations of Cohen’s kappa in agreement studies in ophthalmology

20-08-2024

library(epiR)

## Warning: package 'epiR' was built under R version 4.4.1

Table.1 <- matrix(c(52,6,1,1), nrow = 2, byrow= T)

colnames(Table.1) <- c("Ex2.pass","Ex2.fail")
rownames(Table.1) <- c("Ex1.pass","Ex1.fail")

Table.1

## Ex2.pass Ex2.fail
## Ex1.pass 52 6
## Ex1.fail 1 1

epi.kappa(Table.1)

## $prop.agree
## obs exp
## 1 0.8833333 0.8577778
##
## $pindex
## est se lower upper
## 1 0.85 0.04689429 0.7580889 0.9419111
##
## $bindex
## est se lower upper
## 1 0.08333333 0.04748294 -0.00973152 0.1763982
##
## $pabak
## est lower upper
## 1 0.7666667 0.5485677 0.9035703
##
## $kappa
## est se lower upper
## 1 0.1796875 0.1046165 -0.02535715 0.3847322
##
## $z
## test.statistic p.value
## 1 1.717582 0.08587287
##
## $mcnemar
## test.statistic df p.value
## 1 3.571429 1 0.05878172

Table.2 <- matrix(c(51,0,1,6), nrow = 2, byrow= T)
Table.2

## [,1] [,2]
## [1,] 51 0
## [2,] 1 6

colnames(Table.2) <- c("Ex2.pass","Ex2.fail")
rownames(Table.2) <- c("Ex1.pass","Ex1.fail")
Table.2

## Ex2.pass Ex2.fail
## Ex1.pass 51 0
## Ex1.fail 1 6

epi.kappa(Table.2)

## $prop.agree
## obs exp
## 1 0.9827586 0.8008323
##
## $pindex
## est se lower upper
## 1 0.7758621 0.05855596 0.6610945 0.8906296
##
## $bindex
## est se lower upper
## 1 -0.01724138 0.05855596 -0.1320089 0.09752618
##
## $pabak
## est lower upper
## 1 0.9655172 0.8152779 0.9991272
##
## $kappa
## est se lower upper
## 1 0.9134328 0.1308135 0.6570431 1.169823
##
## $z
## test.statistic p.value
## 1 6.98271 2.895393e-12
##
## $mcnemar
## test.statistic df p.value
## 1 1 1 0.3173105

Table.3 <- matrix(c(27,6,1,26), nrow = 2, byrow= T)
Table.3

## [,1] [,2]
## [1,] 27 6
## [2,] 1 26

colnames(Table.3) <- c("Ex2.pass","Ex2.fail")
rownames(Table.3) <- c("Ex1.pass","Ex1.fail")
Table.3

## Ex2.pass Ex2.fail
## Ex1.pass 27 6
## Ex1.fail 1 26

epi.kappa(Table.3)

## $prop.agree
## obs exp
## 1 0.8833333 0.4966667
##
## $pindex
## est se lower upper
## 1 0.01666667 0.09065094 -0.1610059 0.1943392
##
## $bindex
## est se lower upper
## 1 0.08333333 0.09095685 -0.09493881 0.2616055
##
## $pabak
## est lower upper
## 1 0.7666667 0.5485677 0.9035703
##
## $kappa
## est se lower upper
## 1 0.7682119 0.1273178 0.5186737 1.01775
##
## $z
## test.statistic p.value
## 1 6.033815 1.601331e-09
##
## $mcnemar
## test.statistic df p.value
## 1 3.571429 1 0.05878172

Table.4 <- matrix(c(52,4,3,1), nrow = 2, byrow= T)
Table.4

## [,1] [,2]
## [1,] 52 4
## [2,] 3 1

colnames(Table.4) <- c("Ex2.pass","Ex2.fail")
rownames(Table.4) <- c("Ex1.pass","Ex1.fail")
Table.4

## Ex2.pass Ex2.fail
## Ex1.pass 52 4
## Ex1.fail 3 1

epi.kappa(Table.4)

## $prop.agree
## obs exp
## 1 0.8833333 0.8611111
##
## $pindex
## est se lower upper
## 1 0.85 0.04689429 0.7580889 0.9419111
##
## $bindex
## est se lower upper
## 1 0.01666667 0.04806439 -0.0775378 0.1108711
##
## $pabak
## est lower upper
## 1 0.7666667 0.5485677 0.9035703
##
## $kappa
## est se lower upper
## 1 0.16 0.1281666 -0.09120184 0.4112018
##
## $z
## test.statistic p.value
## 1 1.248376 0.2118936
##
## $mcnemar
## test.statistic df p.value
## 1 0.1428571 1 0.705457

Table.5 <- matrix(c(27,4,3,26), nrow = 2, byrow= T)
Table.5

## [,1] [,2]
## [1,] 27 4
## [2,] 3 26

colnames(Table.5) <- c("Ex2.pass","Ex2.fail")
rownames(Table.5) <- c("Ex1.pass","Ex1.fail")
Table.5

## Ex2.pass Ex2.fail
## Ex1.pass 27 4
## Ex1.fail 3 26

epi.kappa(Table.5)

## $prop.agree
## obs exp
## 1 0.8833333 0.5
##
## $pindex
## est se lower upper
## 1 0.01666667 0.09065094 -0.1610059 0.1943392
##
## $bindex
## est se lower upper
## 1 0.01666667 0.09126173 -0.162203 0.1955364
##
## $pabak
## est lower upper
## 1 0.7666667 0.5485677 0.9035703
##
## $kappa
## est se lower upper
## 1 0.7666667 0.1290277 0.513777 1.019556
##
## $z
## test.statistic p.value
## 1 5.941876 2.817778e-09
##
## $mcnemar
## test.statistic df p.value
## 1 0.1428571 1 0.705457

Table.6 <- matrix(c(28,0,1,27), nrow = 2, byrow= T)
Table.6

## [,1] [,2]
## [1,] 28 0
## [2,] 1 27

colnames(Table.6) <- c("Ex2.pass","Ex2.fail")
rownames(Table.6) <- c("Ex1.pass","Ex1.fail")
Table.6

## Ex2.pass Ex2.fail
## Ex1.pass 28 0
## Ex1.fail 1 27

epi.kappa(Table.6)

## $prop.agree
## obs exp
## 1 0.9821429 0.5
##
## $pindex
## est se lower upper
## 1 0.01785714 0.09446098 -0.167283 0.2029973
##
## $bindex
## est se lower upper
## 1 -0.01785714 0.09446098 -0.2029973 0.167283
##
## $pabak
## est lower upper
## 1 0.9642857 0.8089482 0.999096
##
## $kappa
## est se lower upper
## 1 0.9642857 0.1335454 0.7025416 1.22603
##
## $z
## test.statistic p.value
## 1 7.22066 5.173585e-13
##
## $mcnemar
## test.statistic df p.value
## 1 1 1 0.3173105

set.seed(3456)
r1 <- rbinom(1000,1,0.5)
set.seed(6789)
r2 <- rbinom(1000,1,0.5)

mr <- as.data.frame(cbind(r1,r2))
mr$sum <- r1+r2
mr$sum <- as.factor(mr$sum)
table(mr$sum)

##
## 0 1 2
## 263 503 234

## maxKappa

# 1. Create the original contingency table from your data
# Rows = Rater 1 (OD Breeding), Cols = Rater 2 (OD Breeding)
obs_table <- matrix(c(51, 0,
 1, 6),
 nrow = 2, byrow = TRUE)

# 2. Extract fixed marginal totals
row_totals <- rowSums(obs_table) # Clinician 1: 51 Yes, 7 No
col_totals <- colSums(obs_table) # Clinician 2: 52 Yes, 6 No
n <- sum(obs_table) # Total N = 58

# 3. Create the 'Maximum Agreement' Table (Dunn's Logic)
# Maximize diagonal cells by taking the smaller of the two marginals
a_max <- min(row_totals[1], col_totals[1]) # smaller of 51 and 52
d_max <- min(row_totals[2], col_totals[2]) # smaller of 7 and 6

# Fill in disagreement cells to maintain fixed marginals
b_max <- row_totals[1] - a_max
c_max <- row_totals[2] - d_max

max_table <- matrix(c(a_max, b_max,
 c_max, d_max),
 nrow = 2, byrow = TRUE)

# 4. Calculate Expected Agreement (Pe) based on fixed marginals
pe <- sum((row_totals * col_totals) / n) / n

# 5. Calculate Max Possible Observed Agreement (Po_max)
po_max <- (a_max + d_max) / n

# 6. Final maxK calculation
max_k <- (po_max - pe) / (1 - pe)

# Print Results
cat("--- Maximum Agreement Table ---\n")

## --- Maximum Agreement Table ---

print(max_table)

## [,1] [,2]
## [1,] 51 0
## [2,] 1 6

cat("\nExpected Agreement (Pe):", round(pe, 4))

##
## Expected Agreement (Pe): 0.8008

cat("\nMax Possible Observed Agreement (Po_max):", round(po_max, 4))

##
## Max Possible Observed Agreement (Po_max): 0.9828

cat("\nMaximum Attainable Kappa (maxK):", round(max_k, 4))

##
## Maximum Attainable Kappa (maxK): 0.9134
